# The Research Progress of Tumor‐Associated Macrophages in Prostate Cancer

**DOI:** 10.1155/jimr/6287638

**Published:** 2026-06-04

**Authors:** Si Shen, Zhonghao Tang, Chen Guo, Anjie Chen, Yuanyuan Mi

**Affiliations:** ^1^ Department of Urology, Affiliated Hospital of Jiangnan University, 1000 Hefeng Road, Wuxi, 214122, Jiangsu, China, jiangnan.edu.cn; ^2^ Wuxi School of Medicine, Jiangnan University, 1800 Lihudadao, Wuxi, 214122, Jiangsu, China, jiangnan.edu.cn

**Keywords:** adenosine signaling, immunosuppressive tumor microenvironment, macrophage polarization, prostate cancer, SPP1^hi^-TAMs, tumor-associated macrophages

## Abstract

Prostate cancer (PCa) is a major global cancer burden in men, and its treatment is hindered by the immunosuppressive tumor microenvironment. In this context, PCa initially shows a favorable response to immunotherapy. However, as the disease progresses, the tumor gradually develops resistance to immunotherapy, with tumor‐associated macrophages (TAMs) being key drivers. TAMs promote inflammation, angiogenesis, stromal remodeling, and immune evasion, leading to the development of castration‐resistant PCa. The traditional M1/M2 dichotomy, such as proliferative‐TAM (Prolif‐TAM) and immunoregulatory‐TAM (Reg‐TAM), has been refined by single‐cell RNA sequencing. In this article, we further discuss how signaling pathways regulate TAM polarization and investigate the multidimensional mechanisms by which TAMs drive PCa progression, the pathways that promote immunotherapy resistance, and the role of macrophage extracellular traps (METs) in PCa metastasis. Targeting TAMs for precision treatment of PCa is a promising therapeutic strategy. These strategies include blocking the adenosine pathway to inhibit SPP1^hi^‐TAMs, reprogramming TAMs with immune checkpoint inhibitors (ICIs) to restore chimeric antigen receptor (CAR)‐T cell activity, and novel approaches such as using microRNAs (miRNAs) and natural compounds to modulate TAM polarization. However, these treatment modalities still face challenges, including the complexity of the tumor microenvironment (TME) and TAM heterogeneity, obstacles in drug production and delivery, and toxicity management. This article aims to develop individualized targeting strategies based on the composition, functional status, and signaling dependencies of TAM subpopulations within patient tumors, with the hope of transitioning from a “one‐size‐fits‐all” approach to precision medicine.

## 1. Introduction

As a considerable disease burden among men globally, prostate cancer (PCa) is the leading malignancy in terms of incidence, comprising 31% of new cancer diagnoses and 11% of cancer‐associated mortality based on 2026 data [1]. While localized PCa often has a favorable outcome with standard treatment, the development of mCRPC presents a major therapeutic obstacle, typically resulting in a dismal prognosis despite comprehensive treatment strategies. A key driver of this progression is the immunosuppressive nature of the tumor microenvironment (TME), a process in which tumor‐associated macrophages (TAMs) are pivotal contributors.

TAMs contribute critically to PCa advancement by adopting a spectrum of functional states, historically categorized as M1 (antitumor) or M2 (protumor), which enable them to mold an immunosuppressive TME [2, 3]. Their polarization is precisely calibrated by key pathways like JAK/STAT and NF‐κB, alongside cytokine signals, including CSF‐1 and macrophage migration inhibitory factor (MIF) [3–8]. Recent scRNA‐seq studies have deepened our understanding of TAM diversity, identifying a distinct SPP1^hi^‐TAM population that is particularly abundant in mCRPC. This subset is implicated in immunotherapy resistance by inducing CD8^+^ T cell exhaustion via the adenosine signaling pathway, providing a fresh perspective on immune checkpoint inhibitor (ICI) failure [9].

TAMs orchestrate the malignant progression of PCa via pleiotropic mechanisms. A primary function is immune suppression, achieved through soluble factors (e.g., IL‐10 and TGF‐β) and checkpoint signaling (PD‐1/PD‐L1 and CD47‐SIRPα) [2, 10–13]. They also potentiate metastasis by inducing epithelial‐mesenchymal transition (EMT), remodeling the stroma, and preparing premetastatic niches for circulating tumor cells (CTCs) [14–16]. In parallel, subsets of TAMs contribute to angiogenesis (e.g., via vascular endothelial growth factor A [VEGF‐A] secretion) and metabolic reprogramming, the latter exemplified by lipid‐associated macrophages (LAMs) that foster an immunosuppressive environment [3]. New evidence also implicates macrophage extracellular traps (METs) in metastatic progression, mediated through peptidylarginine deiminase 2 (PAD2)‐dependent activation of CXCL8 signaling [17–19].

Therapeutic interventions targeting TAMs have become a crucial breakthrough point in PCa immunotherapy. Various innovative strategies show potential for reversing immunosuppression: adenosine A2A receptor (A2AR) antagonists (e.g., ciforadenant) specifically block the adenosine signaling of SPP1^hi^‐TAMs, significantly enhancing the efficacy of ICIs [9]; chimeric antigen receptor (CAR)‐T cells combined with PD‐L1 inhibitors eliminate M2‐type TAMs via Fc‐mediated antibody‐dependent cellular phagocytosis (ADCP) and reprogram remaining TAMs towards the M1 phenotype [20]; STING/TLR agonists, delivered via folate receptor‐β (FR‐β)‐targeted nanoparticle systems, enable the in situ reprogramming of TAMs [21, 22]; microRNA (miRNA) modulation (e.g., let‐7b inhibitors) and natural compounds (e.g., omega‐3 fatty acids, tanshinone) regulate TAM functional status by interfering with polarization signaling pathways [23–26].

This review systematically elaborates on the novel classification system, signaling regulatory networks, multidimensional pathogenic mechanisms, and precision targeting strategies of TAMs in PCa. It focuses particularly on the immunosuppressive mechanisms and clinical translational value of cutting‐edge subsets like SPP1^hi^‐TAM, aiming to provide a theoretical basis and practical guidance for overcoming the immunosuppressive TME in mCRPC and optimizing combination treatment strategies.

## 2. Novel Classification System of TAMs

TAMs primarily originate from two pathways: precursor cells during embryonic development and macrophages derived from the infiltration and differentiation of circulating monocytes produced by the bone marrow [18]. Circulating peripheral blood monocytes are the primary recruitment source, while tissue‐resident macrophages contribute minimally [18].

The functional roles of M1 and M2 macrophages within tumors represent a key dichotomy. M1 macrophages, characterized by classical activation, are known for their antitumor functions such as direct cytotoxicity and initiating Th1 immune responses [27]. In contrast, M2 macrophages, arising from alternative activation, exhibit pleiotropic functions that support tumor progression, including promoting immune escape, angiogenesis, and metastasis [27]. This functional spectrum underpins the conventional classification of TAMs into these two broad macrophage dynamics in PCa: molecular to therapeutic insights and phenotypes [2].

However, this traditional model oversimplifies TAM distinctions and fails to explain the vast heterogeneity and plasticity of TAMs, and discrepancies exist with clinical observations. Consequently, recent immunological studies utilize single‐cell RNA sequencing technology for a more refined classification of TAMs, emphasizing multidimensional categorization based on transcriptional signatures and functional states. They are primarily classified into the following five phenotypic categories according to scRNA‐seq analysis: proliferative TAMs (Prolif‐TAMs) [28, 29], immunoregulatory TAMs (Reg‐TAMs) [28], proangiogenic TAMs (Angio‐TAMs) [28], LAMs [28, 30], and tissue‐resident TAMs (RTM‐TAMs) [28].

### 2.1. Prolif‐TAMs (Immunostimulatory TAMs, Prolif‐TAMs)

**Figure 1:**
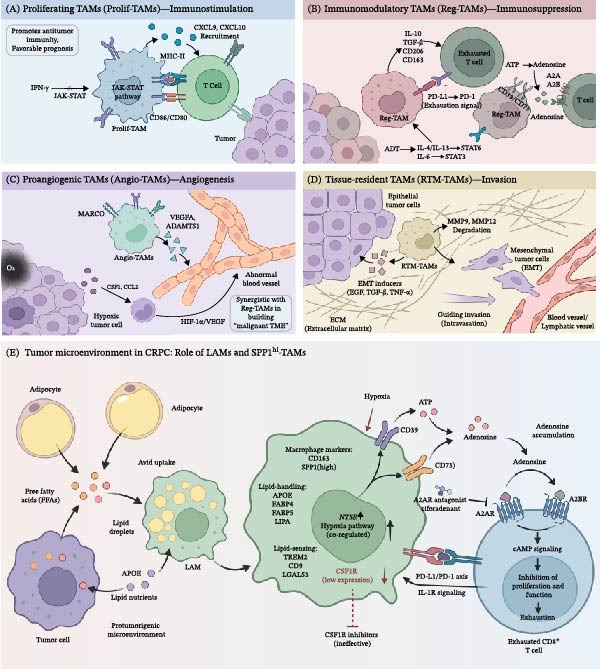
The five main classifications of TAMs and SPP1^hi^‐TAMs: functions and related pathways. (A) Prolif‐TAMs are positively regulated by the IFN‐γ‐activated JAK‐STAT pathway, and promote antitumor responses by secreting cytokines such as CXCL9 and CXCL10 to recruit T cells into the tumor microenvironment. (B) Reg‐TAMs bind to PD‐1 on T cells, blocking T cell activation and ultimately inducing their exhaustion. Furthermore, they can promote the adenosine signaling pathway via high expression of CD39/CD73, leading to immunotherapy resistance. (C) Angio‐TAMs directly stimulate endothelial cell proliferation and promote tumor angiogenesis by highly expressing VEGF‐A and ADAMTS1. Tumors can also recruit monocytes and promote their polarization into an Angio‐TAM phenotype in the vasculature. (D) RTM‐TAMs enhance the motility and invasive potential of tumor cells by degrading the ECM and promoting EMT. (E) LAMs exhibit some functional overlap with SPP1^hi^‐TAMs, sharing similar markers, lipid‐handling genes, and lipid‐sensing receptors. SPP1^hi^‐TAMs can also promote CD8^+^ T cell exhaustion via the adenosine pathway.

Immunostimulatory or Prolif‐TAMs are characterized by the high expression of MHC class II molecules and costimulatory factors (CD86/CD80), which underpin their potent antigen‐presenting capability [31]. This functional phenotype is driven by the upregulation of transcriptional programs governing antigen processing and presentation. Their immunostimulatory role is further mediated through the secretion of cytokines such as CXCL9 and CXCL10, which recruit T cells into the TME and is positively regulated by signaling pathways like IFN‐γ‐activated JAK‐STAT, promoting an antitumor immune response [11, 32]. Clinically, the presence of this TAM subset is associated with the initiation of antitumor immunity and indicates a favorable prognosis in PCa [31] (Figure 1A).

### 2.2. Reg‐TAMs (Immunosuppressive TAMs, Reg‐TAMs)

Reg‐TAMs are key drivers of immune evasion in PCa. They are typically characterized by high expression of immunosuppressive molecules such as IL‐10, TGF‐β, CD206, and CD163, and high surface levels of PD‐L1 that engage with PD‐1 on T cells, transmitting inhibitory signals that block T cell activation and ultimately induce exhaustion [20] (Figure 1B). The differentiation and function of this subset are finely regulated by various signals in the TME. For instance, IL‐4/IL‐13 via the IL‐4Rα‐JAK1/STAT6 axis and elevated IL‐6 after androgen deprivation therapy (ADT) via STAT3 activation significantly promote their immunosuppressive program, which is particularly prominent in the CRPC stage [11, 29, 33]. Furthermore, they can suppress T cell function through the A2A/A2B receptor signaling pathway by catalyzing the conversion of extracellular ATP to adenosine via high expression of ectonucleotidases like CD39/CD73. The enrichment of Reg‐TAMs in mCRPC is closely associated with resistance to ICIs, making them important targets for combination therapy [5, 6].

### 2.3. Angio‐TAMs

Angio‐TAMs are a key cellular population driving the abnormal generation and maturation of the tumor vascular network. This subset directly stimulates endothelial cell proliferation, migration, and tube formation by highly expressing key factors such as VEGF‐A and a disintegrin and metalloproteinase with thrombospondin motifs 1 (ADAMTS1). Its surface marker, MARCO (macrophage receptor with collagenous structure), is not only involved in recognizing apoptotic cells and modified proteins in the TME but is also closely associated with its proangiogenic function [34–36]. Driven by signals such as hypoxia, tumors recruit monocytes through the secretion of chemokines like CSF1 and CCL2 and polarize them into a proangiogenic phenotype via signaling pathways such as HIF‐1α/VEGF [37] (Figure 1C). These newly formed vessels are typically disorganized and dysfunctional, yet they provide the necessary oxygen and nutrients for rapidly growing tumor tissues and serve as channels for the distant metastasis of tumor cells [5, 34].

Different functional TAM subsets do not act in isolation but form a complex collaborative network. Among these, the functional synergy between Angio‐TAMs and Reg‐TAMs is particularly critical for tumor progression [5, 35]. Reg‐TAMs (through the secretion of IL‐10, TGF‐β, high expression of PD‐L1, etc.) shape an immune‐privileged microenvironment that suppresses T cell activity, providing a “sanctuary” for tumor cells [15, 27]. Meanwhile, Angio‐TAMs construct an abnormal vascular network, providing material and energy support for the rapidly proliferating tumor cells and infiltrating immunosuppressive cells in this niche. Together, they coconstruct a “malignant niche” that supports tumor growth, invasion, and metastasis [5, 34, 35]. It is noteworthy that in the stage of mCRPC, the enrichment of Angio‐TAMs is associated with poor prognosis and therapy resistance, making them a potential sensitization target for antiangiogenic therapies (such as those targeting VEGF/VEGFR) [6, 36].

### 2.4. RTM‐TAMs (Proinvasion TAMs, RTM‐TAMs)

RTM‐TAMs are pivotal in facilitating tumor cell dissemination by remodeling the TME. This subset is recruited to the invasive front by tumor‐derived signals (e.g., CCL2) and secretes a repertoire of matrix metalloproteinases (MMPs), notably MMP9 and MMP12, to degrade the extracellular matrix (ECM) and basement membrane, thereby clearing a path for tumor cell invasion [5, 38, 39] (Figure 1D). Beyond physical remodeling, they promote EMT in tumor cells by secreting factors like EGF, TGF‐β, and TNF‐α, enhancing tumor cell motility and invasive potential [11, 27]. Furthermore, they guide disseminated tumor cells towards blood or lymphatic vessels, a process crucial for intravasation, by establishing chemokine (e.g., EGF) and collagen fiber alignment gradients. Their function is often reinforced by cross‐talk with other stromal cells and hypoxic conditions within the tumor core [37, 40].

### 2.5. LAMs

LAMs represent a functionally distinct subset of macrophages within the TME, characterized by profound reprogramming of the lipid metabolism. At the molecular level, LAMs exhibit a unique gene expression profile, encompassing not only typical macrophage markers (e.g., CD163 and SPP1) but also high expression of (Figure 1) lipid‐handing genes (e.g., apolipoprotein E [APOE], FABP4, FABP5, and LIPA) and lipid‐sensing receptors (e.g., TREM2, CD9, and LGALS3) [31–33, 41]. This metabolic state drives massive intracellular lipid droplet accumulation and confers upon them protumor functional features akin to M2 macrophages, including impaired antigen presentation, promotion of angiogenesis, and modulation of the ECM [31, 42] (Figure 1E).

Notably, LAMs overlap to some extent with SPP1^hi^‐TAMs, the latter being considered a functionally specialized immunosuppressive subset within LAMs that collectively mediate immunosuppression via pathways such as adenosine signaling [5, 28]. In PCa, particularly in the CRPC stage, free fatty acids released by tumor cells and adipocytes are avidly taken up by LAMs. This not only sustains their own survival and function but also, through the secretion of apolipoproteins like APOE, provides lipid nutrients to tumor cells, thereby coshaping an immunosuppressive and protumorigenic microenvironment [43, 44].

### 2.6. SPP1^hi^‐TAMs

In malignancies, certain TAM subsets have been discovered that may exhibit mixed functional phenotypes that transcend the boundaries of the classification systems mentioned above. Specific TAM populations might perform specific functions rather than being simply categorized into one of these core classes; therefore, it is important to avoid overinterpreting subtype labels across different tumor types. For example, there is a certain degree of overlap between SPP1^hi^‐TAMs and LAMs. Similar to LAMs, SPP1^hi^‐TAMs often coexpress markers such as TREM2, APOE, and CD9 [9](Figure 1E). Nevertheless, SPP1^hi^‐TAMs are functionally primarily associated with immunotherapy resistance [9], which show a significant difference from LAM. Their numbers within the TME gradually increase with disease progression, and this TAM subset is significantly enriched in mCRPC [9].

Compared to other myeloid cell subsets, SPP1^hi^‐TAMs express more prominent immunosuppressive molecular markers [9]. Additionally, the expression level of SPP1^hi^‐TAM‐related genes strongly correlates with the exhaustion level of CD8^+^ T lymphocytes in tumor‐infiltrated areas, and this exhaustion intensifies with the disease progression. Single‐cell transcriptomics reveals a coregulated hypoxia pathway in SPP1^hi^ ‐TAMs. This drives the upregulation of the ectonucleotidases CD39 and CD73 (encoded by the NT5E gene), a phenomenon particularly prominent in advanced mCRPC, leading to the accumulation of extracellular adenosine. Subsequently, adenosine acts on A2A and A2B receptors expressed on immune cells (including T cells), directly inhibiting their function and proliferation by activating the downstream cAMP signaling pathway.

In vivo studies indicate that in CRPC, SPP1^hi^‐TAMs are a key driver of resistance to ICIs (such as combined anti‐CTLA‐4 and anti‐PD‐1 therapy) [9]. Adoptive transfer of these cells into the CRPC TME significantly impaired the tumor‐suppressive effect of combined ICI therapy, shortened the overall survival (OS) of mice, and simultaneously increased the number of intratumoral exhausted CD8^+^ T cells.

Blocking the adenosine pathway using the A2A receptor antagonist ciforadenant can partially restore T cell function, enhance tumor sensitivity to ICIs, and inhibit tumor growth. However, blocking A2AR alone failed to fully restore T cell proliferation, suggesting that SPP1^hi^‐TAMs may utilize other mechanisms—such as the PD‐1/PD‐L1 axis or IL‐1R signaling pathway—to collectively drive immunotherapy resistance. Furthermore, transcriptome analysis shows that the expression of the CSF1R gene in SPP1^hi^‐TAMs is significantly lower than in other myeloid cells [9], complicating clinical strategies aimed at enhancing ICI efficacy by targeting myeloid cells via CSF1R inhibitors. These results collectively provide strong evidence that SPP1^hi^‐TAMs act as key immune regulators in PCa progression through potent immunosuppression and induction of T cell exhaustion, positioning them as potential drivers of immunotherapy resistance.

## 3. Signaling Regulatory Network of TAM Functional Subsets in PCa

The functional differentiation of TAMs within the PCa TME is not determined by a single signaling pathway but is coregulated by multiple signaling networks operating within a specific pathological context. Unlike the traditional M1/M2 dichotomy, modern single‐cell transcriptomics reveals that TAMs exhibit functional heterogeneity across different stages of PCa progression, encompassing subsets such as proliferative, immunoregulatory, and RTM, as well as specialized subsets like LAM and SPP1^hi^‐TAMs [9, 32]. The formation of these subsets is precisely regulated by core signaling axes like JAK/STAT, NF‐κB, and PI3K/AKT and is profoundly shaped by pathological features characteristic of PCa, such as androgen metabolism, the bone metastasis microenvironment, and progression to castration resistance [3, 5].

### 3.1. The Central Role of the JAK/STAT Signaling Axis in the Differentiation of Immunoregulatory TAMs in PCa

In the PCa microenvironment, the activation status of the JAK/STAT pathway directly determines the functional direction of TAMs. IFN‐γ, via activating the JAK1/JAK2‐STAT1 axis, induces the transcription of genes like iNOS, CXCL9, and CXCL10, driving TAMs towards a proliferative phenotype and enhancing antigen presentation and T cell recruitment capabilities [3, 45]. However, during the progression from localized PCa to mCRPC, significantly elevated levels of IL‐4 and IL‐13 secreted by tumor cells activate the IL‐4Rα‐JAK1/STAT6 pathway. This promotes the nuclear translocation of IRF4, upregulates immunosuppressive molecules like arginase 1 (ARG1) and CD206, and fosters the enrichment of Reg‐TAMs [3, 33]. Notably, the IL‐6 level is significantly increased in the TME following ADT [46]. Sustained IL‐6 signaling activates STAT3, promoting the maintenance of Reg‐TAMs, which is closely associated with resistance to immunotherapy in CRPC.

A hallmark of TAMs in PCa bone metastases is a state of dysregulated STAT3 activation. Triggered by IL‐6/IL‐10 from the microenvironment, the gp130‐JAK‐STAT3 pathway induces immunosuppressive molecules (ARG1 and IL‐10) [47] and facilitates the transactivation of the long noncoding RNA MALAT1, which is linked to bone metastasis [46]. A critical, self‐sustaining mechanism is the ensuing “STAT3‐MALAT1‐CCL2” positive feedback loop: MALAT1 upregulation enhances CCL2 secretion, which in turn recruits more monocytes, ultimately sustaining the TAM population. This process is further refined by metastasis‐specific regulation, wherein mTORC2‐mediated phosphorylation of STAT3 at Ser727—potentiated by the hypoxic, high‐calcium bone niche—augments its DNA‐binding affinity and transcriptional activity [37, 48].

### 3.2. The NF‐κB Pathway and Dynamic Switching of TAM Functional Subsets in PCa

The activation of the NF‐κB pathway in PCa involves an interplay with AR signaling beyond classical inflammatory stimuli. Following ADT, the derepression of NF‐κB due to diminished AR signaling facilitates p65 nuclear translocation [49]. Consequently, NF‐κB induces the expression of monocyte‐recruiting chemokines (e.g., CCL2 and CSF‐1), fostering the differentiation of Reg‐TAMs [6, 34]. Paracrine secretion of IL‐1β and TNF‐α from the activated pathway also promotes EMT in tumor cells, establishing a vicious cycle of TAM‐tumor cell cooperation [16]. In CRPC, the emergence of AR‐V7 and similar variants further potentiates NF‐κB signaling, explaining the elevated infiltration of Reg‐TAMs characteristic of this advanced disease stage [50].

PCa cells possess the capacity to orchestrate premetastatic niche formation in distant sites like the bone by releasing exosomes that modulate resident macrophages. The TLR4 ligands packaged within these exosomes engage the TLR4‐MyD88‐NF‐κB axis in macrophages, prompting the secretion of inflammatory mediators such as CXCL5 and S100A8/A9 [35, 36, 38]. This inflammatory milieu supports CTC seeding, while the educated TAMs further promote osteolytic lesion development by secreting matrix‐degrading enzymes like MMP9 [15]. A key feature in bone metastases is the prevalence of p50/p50 homodimerization of NF‐κB in TAMs, a configuration that favors immunosuppression over inflammation [34].

### 3.3. Synergistic Role of the PI3K/AKT/mTOR Signaling Network in Proangiogenic and Reg‐TAMs

PCa cells highly express CSF‐1, which, upon binding to CSF‐1R on the TAM surface, leads to phosphorylation of the Y721 site, providing a docking site for the PI3K p85 subunit [39]. The activated PI3K‐AKT pathway reprograms TAMs through a dual mechanism: (1) it inhibits the transcription of proinflammatory genes (IL1α, IL1β, and CXCL2) while upregulating ARG1 and IL‐10 [39]; and (2) it induces the expression of miR‐21, which in turn enhances AKT activity by targeting PTEN and PDCD4, forming a positive feedback loop [40]. In mCRPC patients, serum CSF‐1 levels show a significant positive correlation with the extent of immunosuppressive TAM infiltration and are associated with resistance to anti‐PD‐1 therapy [4]. However, transcriptome analysis of SPP1^hi^‐TAMs reveals that their CSF1R expression is significantly lower than that in other myeloid cells [9], suggesting that PI3K/AKT activation in this subset may primarily depend on other upstream signals (such as integrins or growth factor receptors), offering new insights for targeted therapies.

The frequent PTEN loss in PCa causes constitutive activation of the PI3K/AKT/mTOR pathway, which not only promotes tumor cell proliferation but also reshapes the metabolic and functional state of TAMs. mTORC1 activation enhances glycolysis and VEGF‐A secretion in TAMs, driving their differentiation towards a proangiogenic phenotype [51, 52]. Within the hypoxic microenvironment of bone metastases, HIF‐1α synergizes with mTORC1 to upregulate proangiogenic genes like VEGF‐A and MARCO. VEGF‐A secreted by TAMs not only promotes abnormal angiogenesis but also activates PI3K signaling in endothelial cells via VEGFR2, increasing vascular permeability and facilitating tumor cell extravasation [53]. Furthermore, mTORC2 phosphorylates the S473 site of AKT, enhancing its activity and promoting the expression of 14‐3‐3ε and C/EBPβ. These molecules further stabilize the immunosuppressive TAM phenotype by inhibiting the IKK/NF‐κB proinflammatory pathway [54].

### 3.4. Critical Roles of the MAPK Signaling Family in the Differentiation of RTM‐TAM

MIF is highly expressed in PCa and positively correlates with the Gleason score and metastasis risk [55]. MIF binds to the CD74/CXCR4 receptor complex and activates ERK1/2 in a concentration‐dependent manner (peak at 15 ng/mL), with sustained phosphorylation lasting up to 12 h [56, 57]. Activated ERK1/2 not only upregulates characteristic genes (MMP9 and MMP12) of RTM‐TAMs [58] but also phosphorylates the threonine residue of caspase‐9, inhibiting the mitochondrial apoptosis pathway and enhancing TAM survival within the tumor stroma [59]. In a PCa bone metastasis model, MIF‐activated ERK1/2 promotes focal adhesion kinase phosphorylation, strengthening the physical interaction between TAMs and tumor cells [60] and assists CTCs in traversing the endothelial layer and colonizing the bone marrow cavity [61, 62]. The transcriptional repressor HBP1 can directly bind to the MIF promoter (regions from ‐811 to ‐792) to suppress its transcription [63]. However, HBP1 expression is downregulated in advanced PCa, leading to MIF derepression [64], which represents a potential therapeutic intervention point.

The hypoxic microenvironment (oxygen partial pressure <1%) within solid PCa tumors activates p38 MAPK, a key signal for the formation of RTM‐TAMs [65, 66]. Hypoxia‐induced phosphorylation of p38α upregulates HIF‐1α stability, and HIF‐1α synergizes with p38 to transcriptionally activate prometastatic genes such as MMP9 and CXCL8. During the progression to castration‐resistant PCa after ADT, hypoxia within the tumor tissue intensifies [53]. TAMs with sustained p38 MAPK activation secrete large amounts of CXCL5, which recruits neutrophils via the CXCR2 axis, fostering a proinflammatory reaction. This response clears apoptotic tumor cells while simultaneously releasing more proinflammatory factors, forming a vicious cycle of “hypoxia‐p38‐inflammation‐metastasis” [67]. Furthermore, p38 is involved in regulating the cargo of exosomes secreted by TAMs. Under hypoxic conditions, exosomes released by p38‐activated TAMs are enriched with pro‐EMT miRNAs (e.g., miR‐21 and miR‐155), which can be directly transferred to tumor cells to induce a mesenchymal phenotype [16].

### 3.5. Role of the cGAS‐STING Pathway in TAM Functional Reprogramming

The cytosolic DNA‐sensing cGAS‐STING pathway plays a unique role in regulating TAM function. In PCa, DNA fragments released by tumor cell death induced by radiotherapy or chemotherapy activate the cGAS in TAMs, leading to the synthesis of cGAMP. cGAMP binds to STING, initiating dual signaling tracks involving TBK1‐IRF3 and IKK‐NF‐κB [68]. STING activation can reprogram Reg‐TAMs towards a proliferative phenotype: upregulating antigen‐presenting molecules like CD80, CD86, and MHC II and secreting CXCL9 and CXCL10 to recruit CD8^+^ T cells [69]. In a PCa mouse model combining ADT with a STING agonist, perivascular Reg‐TAMs were significantly reduced, and tumor growth was delayed [22]. Mechanistically, STING‐activated IRF3 inhibits Tyr705 phosphorylation of STAT3, blocking the IL‐6‐induced immunosuppressive program. Concurrently, NF‐κB‐mediated production of type I interferons activates the downstream JAK1‐STAT1 pathway, establishing a proinflammatory signal that antagonizes STAT6 signaling [70].

### 3.6. The Central Role of Lipid Metabolic Reprogramming in the Differentiation of LAM and SPP1^hi^‐TAMs

LAM and SPP1^hi^‐TAMs share the characteristic of coexpressing high levels of lipid metabolism genes (such as APOE, FABP4, FABP5, and LPL) and lipid receptors (like TREM2 and CD36) [32]. In the PCa microenvironment, free fatty acids released by tumor cells and adipocytes are taken up by TAMs and activate peroxisome proliferator‐activated receptor gamma (PPARγ). This, in turn, upregulates fatty acid‐binding proteins and cholesterol transport proteins [71]. Concurrently, the hypoxic and nutrient‐stressed TME activates AMPK, which inhibits de novo fatty acid synthesis by phosphorylating acetyl‐CoA carboxylase [43]. However, in the mCRPC stage, regulatory T cells (Tregs), by suppressing IFN‐γ production from CD8^+^ T cells, relieve the inhibition on sterol regulatory element‐binding protein 1 (SREBP1) [72]. This restoration of fatty acid synthesis promotes lipid droplet accumulation in LAMs/SPP1^hi^‐TAMs. Lipid‐laden TAMs not only exhibit reduced antigen‐presenting capacity but also secrete APOE and SPP1, which enhance the lipid uptake and membrane fluidity of tumor cells, thereby increasing their invasive potential [73]. The reprogramming of lipid metabolism in TAMs, including the upregulation of lipogenesis mediated by factors like SREBP1, is increasingly recognized as a critical regulator of macrophage polarization and function within the TME. Targeting these metabolic pathways, such as by inhibiting key enzymes in fatty acid synthesis, presents a potential strategy to counteract the protumor functions of these macrophage subsets.

### 3.7. Evolution of the CCL2‐CCR2 Axis in TAM Recruitment Across Different Stages of PCa

In hormone‐sensitive PCa, activation of the AR upregulates the epigenetic modifier enzyme EZH2. EZH2, in turn, represses the transcription of miR‐124‐3p via H3K27me3 modification [44]. The loss of miR‐124‐3p relieves its posttranscriptional inhibition on the CCL2 3^′^ untranslated region (3’UTR), leading to substantial secretion of CCL2, which recruits CCR2^+^ monocytes [6]. These newly recruited monocytes differentiate into Reg‐TAMs under the synergistic action of IL‐6 and CCL2 in the TME, a process involving STAT3 activation [50]. After ADT, although AR signaling is suppressed, tumor cells maintain low expression of miR‐124‐3p by upregulating AR‐independent expression of EZH2 (e.g., via the E2F transcription factor) [74, 75], resulting in persistently high secretion of CCL2. This mechanism explains why TAM infiltration continues to increase in CRPC patients even under very low‐androgen conditions.

## 4. Integration of Multidimensional Mechanisms by Which TAM Functional Subsets Drive PCa Progression

The progression of PCa from a localized disease to mCRPC is a multistep process driven by dynamic alterations in the TME [31]. Distinct functional subsets of TAMs play a central role in key steps such as inflammation initiation, vascular remodeling, stromal modification, and immune escape. Contrary to the traditional view of TAMs as a single protumor entity, modern single‐cell transcriptomics reveals the spatiotemporal distribution patterns and specific pathogenic mechanisms of various TAM functional subsets across different stages of PCa development. The dysregulation of the dynamic balance between these subsets is a key driver of malignant disease progression.

### 4.1. The Initiating Role of the Chronic Inflammation‐TAM Axis in Prostate Carcinogenesis

**Figure 2:**
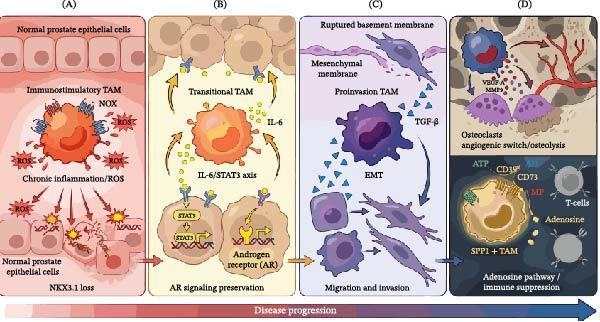
The evolving role of TAMs in PCa: from initiation to metastasis. (A) Immunostimulatory TAMs (proliferative TAMs) upregulate NOX, releasing large amounts of ROS that induce the degradation of NKX3.1, thereby damaging the DNA of normal prostate epithelial cells. (B) Chronic inflammation and transitional TAMs (such as immunoregulatory TAMs) synergistically release IL‐6, which upregulates the expression of the androgen receptor (AR) and its variant AR‐V7 via the STAT3 pathway, thereby enabling tumor cells to acquire androgen‐independent growth ability. (C) TAMs highly express and secrete numerous EMT‐inducing factors, such as TGF‐β, which promote the EMT process. This leads to the loss of intercellular connections in tumor cells and enhances their migratory ability. (D) At the osteolytic lesion stage, proangiogenic TAMs secrete VEGF‐A and MMP9, which interact to promote osteolysis and ultimately stimulate tumor cell proliferation；SPP1^hi^‐TAMs highly express the ectonucleotidases CD39 and CD73. CD39 hydrolyzes extracellular ATP/ADP to AMP, which is subsequently converted by CD73 into adenosine. The resulting adenosine binds to the A2A and A2B receptors on T cells, activating the Gs protein–adenylate cyclase–cAMP signaling pathway and thereby inducing multiple immunosuppressive effects.

The occurrence of PCa is associated with chronic inflammation, wherein the functional dysregulation of Prolif‐TAMs is a critical step in disease initiation. In inflammatory microenvironments induced by chronic bacterial prostatitis or a high‐fat diet, infiltrating Prolif‐TAMs upregulate NADPH oxidases (NOX1, NOX2, and NOX4), generating large amounts of reactive oxygen species (ROS); local ROS levels can be 3–5 times higher than in normal prostate tissue [76, 77]. Persistent oxidative stress can directly damage the DNA of prostate epithelial cells and also induce proteasomal degradation of the tumor suppressor NKX3.1 by activating stress‐response genes [78, 79] (Figure 2A). The loss of NKX3.1 impairs its transcriptional regulation of DNA repair enzymes (such as ATM and BRCA1) and its ability to counteract oxidative damage, thereby leading to the accumulation of genomic instability. Histologically, oxidative damage promotes the formation of proliferative inflammatory atrophy (PIA), a lesion often adjacent to or fused with prostatic intraepithelial neoplasia (PIN), suggesting that PIA may be a precancerous lesion for PCa.

During this period, Prolif‐TAMs exhibit a functional paradox. On the one hand, they release proinflammatory factors such as TNF‐α and IL‐1β to activate the NF‐κB signaling in epithelial cells, upregulating antiapoptotic proteins like Bcl‐2 and cIAP to protect damaged cells from apoptotic clearance. On the other hand, sustained inflammatory signaling leads to epigenetic modifications, such as DNA methylation in promoter regions, causing inflammation response genes (e.g., IL‐6 and IL‐8) to remain elevated and fail to downregulate promptly, creating an “inflammatory memory” [80, 81]. This chronic low‐grade inflammatory state provides continuous mutagenic pressure for subsequent malignant transformation. It is important to note that during the transition from PIA to PIN, Prolif‐TAMs are gradually eliminated, while Reg‐TAMs begin to accumulate. The molecular mechanism underlying this subset switch may be associated with the activation of the HIF‐1α‐STAT3 axis triggered by local hypoxia and the accumulation of metabolites such as lactate.

AR signaling has a dual role in shaping the inflammatory microenvironment of PCa. During the hormone‐sensitive stage, androgens promote the transcription of immunosuppressive factors like IL‐10 and TGF‐β via the AR while reducing the secretion of the proinflammatory factor IL‐6, achieving a state of relative immune homeostasis [82]. However, proinflammatory factors released during chronic inflammation, particularly IL‐6, can enhance the transcriptional activity of AR via the STAT3 pathway, maintaining AR signaling even in a low‐androgen environment. This is a key reason for disease recurrence after ADT [83]. More critically, inflammation‐induced increased expression of aromatase (CYP19A1) in TAMs converts adrenal‐derived androgen precursors (such as DHEA) into active androgens, locally creating an “androgen microenvironment”. This explains why sufficient androgen concentrations to activate AR can still be detected in PCa tissues after ADT.

As castration‐resistant PCa develops, transitional TAMs including immunoregulatory types gradually gain the upper hand. IL‐6 and IL‐8 secreted by TAMs promote the expression of AR splice variants (e.g., AR‐V7) by activating STAT3, enabling tumor cells to achieve androgen‐independent growth (Figure 2B). Simultaneously, they upregulate immune checkpoint molecules like PD‐L1, suppressing the remaining antitumor immunity [84]. Furthermore, metabolic reprogramming in the TME after ADT (enhanced glycolysis and inhibited fatty acid oxidation) leads to further enrichment of Reg‐TAMs and LAMs, forming a vicious cycle of “androgen deprivation–metabolic reprogramming–immunosuppressive TAM enrichment” [53, 85]. This cascade reaction is not confined to the primary site; it propagates inflammatory and immunosuppressive signals to distant organs via circulating exosomes, preparing the “soil” for the formation of distant metastases [86, 87].

### 4.2. RTM‐TAM‐Mediated Tumor Metastatic Cascade

EMT is a crucial process through which tumor cells acquire invasive and migratory capabilities, and RTM‐TAM are primary inducer of EMT. These TAMs highly express and secrete various EMT‐inducing factors, notably TGF‐β, TNF‐α, and IL‐1β. TGF‐β activates the Smad2/3 signaling pathway within tumor cells, leading to transcriptional repression of the epithelial marker E‐cadherin and upregulation of mesenchymal markers like vimentin and N‐cadherin. This results in the loss of intercellular junctions and confers motility to tumor cells (Figure 2C). TNF‐α and IL‐1β contribute by activating transcription factors such as NF‐κB and AP‐1, which subsequently upregulate EMT‐related transcription factors (e.g., Snail, Slug, and Twist). In PCa, EMT features are observed not only at the invasive front of the primary tumor but are also commonly detected in tumor cells that persist after ADT, associating this process with therapy resistance and recurrence.

A bidirectional paracrine network exists between RTM‐TAM and tumor cells. Tumor cell‐derived CSF‐1 recruits and sustains the survival of these TAMs, while TAM‐secreted EGF promotes the chemotactic movement of tumor cells. This reciprocal interaction leads to the formation of “TAM‐tumor cell clusters” at the tumor invasion front that migrate collectively towards blood vessels. Intravital imaging studies have shown prolonged direct contact between tumor cells and TAMs at the invasive front. TAMs secrete MMP9 and MMP12 that degrade the ECM, thereby creating migration paths for tumor cells. MMP9 specifically degrades type IV collagen (a major component of the basement membrane) and cleaves ECM‐sequestered growth factors (e.g., VEGF precursors and latent TGF‐β complexes), releasing their active forms and amplifying pro‐invasive signals. In PCa bone metastasis models, the abundance of type I collagen in the bone matrix provides a rich substrate for MMP9. The collagen degradation mediated by TAMs releases calcium ions and growth factors, collectively forming “chemoattractants” that shape the bone metastatic microenvironment.

Intravasation, the entry of tumor cells into the bloodstream, is a critical bottleneck step in metastasis, and RTM‐TAM facilitate this process through multiple mechanisms. At the vessel wall, TAM‐derived MMP9 degrades the subendothelial basement membrane, increasing vascular permeability. Concurrently, integrin α4β1 expressed on TAMs interacts with VCAM‐1 on the surface of endothelial cells, inducing endothelial cell retraction, exposing the basement membrane, and providing a passage for tumor cells to traverse. Intriguingly, intravital imaging reveals that some TAMs extend pseudopods to physically “push” tumor cells through the endothelial layer, a process requiring the actin cytoskeleton rearrangement and generation of contractile force within the TAMs. CTCs face threats like shear stress and anoikis. TAMs associated with the surface of CTCs express integrin α4, which facilitates binding to platelets, forming “CTC‐TAM‐platelet” aggregates. These aggregates provide physical protection to CTCs and enhance their adhesion potential in distant organs.

The preparation of the premetastatic niche begins at the primary site. Exosomes released by tumor cells, carrying chemokines (CCL2 and CXCL5) and proinflammatory factors (S100A8/A9), travel to future metastatic sites (e.g., bone marrow and lung). There, they activate resident macrophages, prompting them to release more chemokines and establish a chemotactic gradient that recruits CTCs back to the site. These “educated” macrophages in the bone marrow preemptively degrade the bone matrix, creating physical space for CTCs. They also upregulate VEGF and CXCL12 to promote angiogenesis and recruit myeloid‐derived suppressor cells, thereby fostering an immunosuppressive microenvironment. The levels of CCL2 and CXCL5 in circulating exosomes show a significant correlation with the risk of bone metastasis in PCa patients, suggesting their potential utility as predictive biomarkers. The activation window for the premetastatic niche can open months before the diagnosis of the primary tumor, providing a theoretical basis for early intervention strategies.

### 4.3. Pathological Link Between Angio‐TAMs and Abnormal Vascular Network Formation

During the rapid growth of PCa solid tumors, the supply of oxygen and nutrients fails to meet demand, resulting in a hypoxic microenvironment (oxygen partial pressure <1%), which enriches Angio‐TAMs. Under hypoxia, HIF‐1α stabilizes and forms a heterodimer with HIF‐1β, activating a cluster of proangiogenic genes, with VEGF‐A being a primary effector molecule [53]. Angio‐TAMs are a major source of VEGF‐A within the tumor, secreting VEGF‐A at concentrations 2–3 times higher than surrounding tumor cells. VEGF‐A binding to the VEGFR2 receptor on endothelial cells activates downstream PI3K/AKT and ERK1/2 signaling pathways, promoting endothelial cell proliferation, migration, and tube formation. However, the neovasculature formed within tumors is structurally abnormal, characterized by incomplete vessel walls, absent basement membranes, irregular diameters, and severe leakage. This aberrant vascular network paradoxically exacerbates intratumoral hypoxia and interstitial hypertension [88].

Spatially, Angio‐TAMs are notably concentrated in specific regions, particularly the hypoxic‐vascular interface at the tumor margin, rather than the necrotic core. This positioning allows them to sense both hypoxic signals and nascent blood vessels, establishing a positive feedback loop of hypoxia sensing, VEGF secretion, angiogenesis, and further TAM recruitment. Beyond VEGF‐A, Angio‐TAMs also highly express MARCO (a scavenger receptor) and PDGF. MARCO helps clear apoptotic endothelial cells to maintain vascular stability, while PDGF recruits perivascular smooth muscle cells to participate in vessel maturation [89]. However, excessive PDGF signaling in the PCa microenvironment leads to incomplete pericyte coverage, further aggravating vascular abnormalities. Functionally, Angio‐TAMs cooperate with Reg‐TAMs. The latter secrete IL‐10 and TGF‐β, which not only suppress T cells but also upregulate PD‐L1 expression on endothelial cells, forming a “vascular immune barrier” that impedes T cell extravasation [90].

The pattern of angiogenesis in PCa bone metastases differs significantly from that in the primary site, largely due to the unique bone marrow microenvironment. Within bone metastases, Angio‐TAMs not only sustain the tumor’s blood supply but also participate in the coupling of osteolysis and osteogenesis through a “angiogenesis–osteogenesis coupling” mechanism. During the osteolytic lesion phase, Angio‐TAMs secrete VEGF‐A and MMP9 (Figure 2D), which interact synergistically: VEGF‐A promotes the migration and differentiation of osteoclast precursors, while MMP9 degrades the bone matrix, releasing sequestered growth factors (e.g., TGF‐β and IGF‐1). These growth factors, in turn, stimulate tumor cell proliferation, creating a “vicious cycle” [91]. Intriguingly, in some PCas (particularly post‐ADT), lesions can be osteoblastic. In this context, Angio‐TAMs secrete BMP‐2 and Wnt ligands to promote osteoblast differentiation, forming an abnormally mineralized bone matrix that encases tumor cells, providing physical protection and isolating them from chemotherapeutic agents [8].

The metabolic profile of Angio‐TAMs in bone metastases, also undergoes changes. Compared to their counterparts in the primary tumor, the high‐calcium environment within the bone marrow activates the calcium‐sensing receptor on TAMs, promoting cAMP‐PKA signaling that enhances HIF‐1α transcriptional activity. This allows TAMs to maintain a proangiogenic phenotype even under a relatively higher oxygen partial pressure (~5%). Furthermore, CXCL12 secreted by bone marrow stromal cells recruits CXCR4‐positive TAM precursors via the CXCR4 receptor, driving their differentiation towards a proangiogenic phenotype. This axis is critically important for establishing the early bone metastatic niche [92]. Antiangiogenic therapies targeting VEGF‐A (e.g., bevacizumab) have shown limited efficacy in PCa bone metastases. Compensatory upregulation of alternative proangiogenic factors (e.g., FGF2 and Ang2) within the bone marrow microenvironment is a key reason for this poor response, underscoring the need for multitargeted combination blockade strategies.

### 4.4. Special Pathogenic Roles of SPP1^hi^‐TAMs and LAMs in Immune Evasion

LAMs contribute to immune evasion largely due to functional impairments caused by lipid overload. LAMs highly express fatty acid transport proteins (e.g., FATP1 and CD36) and fatty acid‐binding proteins (e.g., FABP4 and FABP5), leading to massive uptake of free fatty acids and oxidized low‐density lipoprotein (oxLDL) from the TME and consequent intracellular lipid droplet accumulation. Lipid overload triggers endoplasmic reticulum stress, activating the PERK‐eIF2α‐ATF4 pathway, which upregulates proapoptotic molecules like CHOP while also inducing protective autophagy, allowing LAM survival under stress. Chronic lipid loading causes LAM dysfunction manifested as (1) impaired antigen‐presenting capacity, as lipids disrupt the proper loading of MHC‐II molecules in endosomes and their trafficking to the cell surface, reducing effective CD4^+^ T cell activation; (2) reduced phagocytic ability, as lipid droplets occupy cytoplasmic space and interfere with phagosome–lysosome fusion, decreasing the clearance of apoptotic tumor cells; and (3) a shift towards an immunosuppressive phenotype, characterized by reduced proinflammatory cytokine production and increased anti‐inflammatory factors like IL‐10.

LAMs play a particularly significant role in PCa bone metastasis. Bone marrow is a lipid‐rich organ, and marrow adiposity increases after ADT, providing ample lipid sources for LAMs. Bone marrow LAMs are not only immunosuppressive themselves but also secrete APOE to enhance lipid uptake by tumor cells, which utilize these fatty acids for β‐oxidation (energy production) and membrane lipid synthesis to support rapid proliferation. Furthermore, TREM2, a lipid‐sensing receptor expressed by LAMs, recognizes phosphatidylserine (a marker on apoptotic cell membranes). While involved in phagocytosis, TREM2 signaling also inhibits proinflammatory signaling, thereby reinforcing the immunosuppressive microenvironment.

SPP1^hi^‐TAMs are a characteristic subset of LAM, and their immunosuppressive function is primarily executed through the adenosine signaling axis. Under hypoxic conditions, SPP1^hi^‐TAMs highly express the ectonucleotidases CD39 and CD73 (Figure 2D). CD39 hydrolyzes extracellular ATP/ADP into AMP, which is then converted to adenosine by CD73. The adenosine concentration in the TME can reach 10–100 μM, significantly higher than in the normal tissue (<1 μM). Adenosine binding to A2A and A2B receptors on T cells activates the Gs protein‐adenylyl cyclase‐cAMP signaling pathway, leading to multiple immunosuppressive effects: (1) inhibition of ZAP70 and Lck kinase activity downstream of the T cell receptor (TCR), preventing T cell activation; (2) upregulation of FoxP3 expression, promoting the differentiation of Tregs; (3) reduction in IFN‐γ and granzyme B production, impairing the cytotoxicity of CD8^+^ T cells; and (4) induction of exhaustion markers (PD‐1, TIM‐3, and LAG‐3) on T cells, accelerating T cell functional inactivation.

The transcriptional signature of SPP1^hi^‐TAMs indicates that, beyond the adenosine pathway, this subset co‐opts other immunosuppressive mechanisms. Single‐cell RNA sequencing reveals high expression of PD‐L1, CD80 (a ligand for CTLA‐4), and IDO1 (an enzyme metabolizing tryptophan to the immunosuppressive metabolite kynurenine) in SPP1^hi^‐TAMs, constituting a “triple immune checkpoint blockade” mechanism. This helps explain the limited efficacy of single‐agent PD‐1 or CTLA‐4 inhibitors in mCRPC. The osteopontin (SPP1) secreted by SPP1^hi^‐TAMs is multifunctional: it binds to integrin αvβ3 on tumor cells to promote invasion and metastasis; interacts with CD44, activating Src and ERK signaling to enhance tumor cell survival; and in bone metastases, recruits osteoclast precursors to promote bone resorption. The coordinated protumor and immunosuppressive actions of SPP1^hi^‐TAMs position them as a critical nexus linking tumor progression and immune escape. Interestingly, LAMs and SPP1^hi^‐TAMs show significant transcriptional overlap but are not identical—the SPP1 signature primarily reflects immunosuppressive function, whereas the LAM signature is dominated by metabolic characteristics. This suggests that they may represent different maturation stages or functional states within a lipid metabolism‐driven TAM differentiation spectrum.

## 5. The Emerging Role of METs in PCa Metastasis

METs are reticular DNA–protein complex structures released by macrophages, representing a novel form of cell death termed METosis. Similar to neutrophil extracellular traps (NETs), METs are composed of decondensed chromatin DNA scaffolds, histones (H1, H2A, H2B, H3, and H4), and various cytoplasmic proteins (myeloperoxidase, elastase, and MMPs). Their primary function is to trap and kill pathogens physically and via proteases [17, 19].

Although research on METs in PCa is still in its early stages, findings in pancreatic and colon cancers, which share biological similarities with PCa, provide insights into the role of METs in PCa. METs have been identified as an independent poor prognostic factor, significantly associated with postoperative recurrence, distant metastasis, and shorter recurrence‐free survival, possessing an independent predictive value [18, 93]. Mechanistic studies reveal that METs promote metastasis by activating the CXCL8‐CXCR1/2 signaling pathway: proteases (e.g., elastase and MMPs) released from METs cleave and activate the precursor form of CXCL8. Active CXCL8 enhances the adhesion capacity of tumor cells and vascular endothelial cells by 2–3 times, a critical step in tumor cell extravasation [94, 95]. CXCL8 also has dual roles in promoting angiogenesis and tumor cell migration, acting at multiple stages of the metastatic cascade. Furthermore, METs exhibit dual effects in the TME similar to NETs: first, by creating a physical barrier—the DNA–protein network can block effector T cells and NK cells from infiltrating the tumor nest, thereby protecting tumor cells from immune attack [96]; second, by emitting protumor signals—MMPs (e.g., MMP9 and MMP12) present in METs degrade the ECM and activate latent growth factors (e.g., TGF‐β), enhancing tumor invasiveness and metastatic potential [96].

The molecular pathways of MET formation primarily rely on NOX [97] and PAD pathways [98, 99]. Among these, PAD2 is identified as a key regulatory enzyme in MET formation, making pharmacological inhibition of PAD2 a promising therapeutic strategy for suppressing tumor metastasis. In a colon cancer liver metastasis model, treatment with the PAD2‐specific inhibitor PAD2‐IN‐1 significantly reduced MET occurrence, effectively decreased tumor cell metastatic capability in vivo, and lowered the level of macrophage infiltration in liver metastases [18]. However, direct research targeting the METs‐PAD2 axis in PCa remains limited. Considering the high incidence of PCa bone metastasis and the abundance of macrophages in the bone marrow, PAD2 inhibitors may have potential in preventing the preparation of the premetastatic niche in bone and inhibiting established metastases. It is important to note that PAD inhibition requires consideration of selectivity: PAD4 is predominantly expressed in neutrophils and involved in NET formation, whereas PAD2 is dominant in macrophages. Specific inhibition of PAD2 could minimize the impact on neutrophil function. Additionally, the potential of METs as biomarkers warrants attention; for example, levels of circulating MET markers (e.g., citrullinated histone H3 and extracellular DNA–MPO complexes) correlate with postoperative recurrence risk in pancreatic cancer [93]. Developing METs detection methods for PCa (e.g., liquid biopsy assays for circulating METs components) could provide new tools for early identification of high‐risk metastatic patients and monitoring treatment response.

Notably, other immune cells, immune molecules, and immunogenic cell death processes may also influence the patient prognosis. Current research lacks mechanistic exploration of the interactions between NETs, METs, and other extracellular traps from immune cells [93]. Furthermore, future investigations into PCa treatment strategies could focus on combination therapies, integrating METs targeting with immunotherapy (e.g., anti‐PD‐1/PD‐L1). Theoretically, this could simultaneously disrupt the physical barrier and immune checkpoint inhibition, enhancing T cell infiltration and tumor killing.

## 6. TAM Functional Subsets as Biomarkers for Prognostic Assessment and Treatment Response in PCa

### 6.1. Prognostic Value and Prediction of Therapy Resistance by Reg‐TAMs and SPP1^hi^‐TAMs

While overall TAM infiltration is associated with a poor prognosis in PCa patients, the prognostic significance varies considerably among different functional subsets. Traditional immunohistochemistry studies have shown that high density of CD68^+^ TAM infiltration (≥22 cells/high‐power field) correlates significantly with reduced cancer‐specific survival and relapse‐free survival [100]. However, CD68 serves as a pan‐macrophage marker and cannot distinguish between functional subsets, limiting its predictive accuracy. Further analyses reveal that CD163^+^ M2‐like TAMs, a classic marker for Reg‐TAMs, are a stronger independent prognostic factor than total TAMs. In mCRPC patients, high infiltration of CD163^+^ TAMs is associated with shorter OS and higher Gleason scores [92, 101]. This association is observed not only in primary tumors but also in bone and lymph node metastases, indicating that Reg‐TAMs promote tumor progression throughout the disease course.

SPP1^hi^‐TAMs, a novel subset identified by single‐cell transcriptomics, are garnering increasing attention for their prognostic and predictive value. Cohort studies of PCa patients at different stages show that the abundance of SPP1^hi^‐TAMs increases stepwise with disease progression: from 5% to 10% in localized PCa to 30%–40% in mCRPC and is closely associated with primary resistance to ICI therapy [9]. A gene signature expression score for SPP1^hi^‐TAMs, calculated from bulk RNA sequencing data, can independently predict the response to ICI treatment. In mCRPC patients receiving combined anti‐CTLA‐4 and anti‐PD‐1 therapy, the objective response rate (ORR) was significantly lower in the high SPP1^hi^‐score group compared to the low‐score group (8% vs. 35%, *p*  < 0.01), and the median progression‐free survival was nearly 50% shorter [9]. This finding provides a molecular basis for patient selection for ICI therapy and informs combination strategies. Furthermore, the abundance of SPP1^hi^‐TAMs shows a significant positive correlation with the extent of CD8^+^ T cell exhaustion within the tumor (*r* = 0.68, *p*  < 0.001), reinforcing its role as a key driver of the immunosuppressive microenvironment.

### 6.2. Association of Angio‐TAMs With Response to Antiangiogenic Therapy

The Angio‐TAM infiltration pattern is associated with tumor microvessel density (MVD) and the degree of vascular abnormality. In PCa, the hallmark subset of Angio‐TAMs is mainly concentrated at the invasive front of the tumor margin and in perivascular areas. Angio‐TAMs (CM‐M2) positively correlate with tumor angiogenesis: CM‐M2 significantly promotes either human umbilical vein endothelial cells (driving de novo angiogenesis) or endothelial progenitor cells (correlated with vasculogenesis) [91]. High infiltration of Angio‐TAMs predicts a higher risk of metastasis and is also associated with resistance to antiangiogenic therapy. In baseline tumor specimens, the overall number of TAMs is an effective predictor of treatment response in mCRPC patients receiving cabozantinib: early responders to anti‐PD‐1 therapy exhibit a higher frequency of TAMs compared to late progressors with poor treatment response [102]. Cabozantinib (anti‐PD‐1 drug) inhibits angiogenesis directly by targeting receptors like VEGFR2 [103] and MET [104] and also has immunomodulatory effects—downregulating proinflammatory M1‐like TAMs and inducing M2‐like TAM polarization. This paradoxical effect can suppress tumor angiogenesis and remodel the immunosuppressive TME, thereby limiting metastasis.

The prognostic significance of TAMs is even more pronounced in the context of bone metastasis. The extent of TAM infiltration within bone metastatic lesions positively correlates with the severity of osteolytic lesions and the incidence of skeletal‐related events [91], such as pathological fractures and spinal cord compression. CXCL5, a chemokine secreted by TAMs when clearing necrotic tumor cells, is significantly elevated in the serum of patients with bone metastases and is associated with shorter OS [67]. Circulating TAM‐related factors could serve as liquid biopsy biomarkers. METs markers, like circulating citrullinated histone H3, are significantly elevated in metabolically active bone metastases and correlate with the number of metastases and the speed of disease progression [105]. Although research on METs markers in PCa is limited, findings in other solid tumors suggest potential translational value.

### 6.3. Synergistic Predictive Role of PD‐L1 Expression Dynamics and TAM Subsets

While PD‐L1 expression on tumor and immune cells (especially TAMs) is a well‐established biomarker for ICI response, its prognostic utility in PCa is inconsistent, largely attributable to spatiotemporal heterogeneity. Initial expression is typically low (<5% positivity) in hormone‐sensitive disease but becomes upregulated after ADT on both malignant cells and TAMs, a process potentially driven by IFN‐γ and IL‐6 [84, 106]. Critically, PD‐L1 expression on TAMs is subset‐specific: it is predominantly high on immunosuppressive subsets like CD163^+^ and SPP1^hi^‐TAMs but low on immunostimulatory MHCII^hi^ CD80^+^ TAMs.

In the context of immunotherapy, evaluating tumor cell PD‐L1 alone is insufficient for predicting the response in PCa patients. Combined analysis shows that predictive power improves significantly when considering both PD‐L1 expression and SPP1^hi^‐TAM abundance. In a cohort treated with ipilimumab (anti‐CTLA‐4), patients with PD‐L1‐positive tumors but low SPP1^hi^‐TAM abundance had an ORR of 45%, whereas those with both PD‐L1 positivity and high SPP1^hi^‐TAM abundance had an ORR of only 12% [107]. The immunosuppression mediated by SPP1^hi^‐TAMs involves pathways independent of PD‐1/PD‐L1, such as the adenosine signaling axis, which is a key reason for the inefficacy of PD‐1 inhibitors alone. Furthermore, after ipilimumab treatment, both CD4^+^ T cells and CD68^+^ TAMs increase within the tumor, but the increase in M2‐subtype TAMs is more pronounced [107]. This “paradoxical immune activation” may represent an adaptive response of the tumor to ICIs, potentially involving upregulation of the IL‐6‐STAT3 axis to recruit more Reg‐TAMs to counteract the treatment effect. This highlights the limitations of ICI monotherapy and provides a rationale for combination strategies targeting TAM recruitment or reprogramming.

### 6.4. Regulatory Role of the CD47‐SIRPα Axis in TAM Phagocytic Function and Treatment Response

The interaction between the ubiquitously expressed cell surface protein CD47 and its receptor SIRPα on macrophages delivers a potent “don’t eat me” signal that suppresses phagocytosis [12]. In PCa, tumor cells exhibit a 3–5‐fold upregulation of CD47 compared to normal epithelium, facilitating immune evasion by thwarting clearance by TAMs [108, 109]. This CD47 overexpression is clinically significant, showing positive associations with higher Gleason scores, advanced clinical stage, and increased risk of biochemical recurrence, marking it as a prognostic biomarker. Notably, the oncogene c‐Myc, frequently dysregulated in PCa, transcriptionally regulates CD47, potentially enabling tumor cells to sustain this high level of immune‐inhibitory signaling without inducing apoptosis [110].

## 7. Precision Targeting Strategies for TAM Functional Subsets in PCa

The treatment of advanced PCa, particularly mCRPC, remains challenging. Although the emergence of various therapies—such as docetaxel chemotherapy, novel androgen receptor antagonists like enzalutamide, and radium‐223 radiopharmaceutical therapy—has extended patient survival, nearly all patients eventually develop resistance. Beyond significant side effects (e.g., myelosuppression, fatigue, and hepatotoxicity), their impact on the immune microenvironment is often overlooked. Chemotherapy‐induced cell death can recruit more Reg‐TAMs, and while radiotherapy may activate immune responses, it can also upregulate checkpoint molecules like PD‐L1. Therefore, developing novel strategies that simultaneously enhance the efficacy of existing treatments and remodel the immunosuppressive TME is critical for improving outcomes in advanced PCa.

### 7.1. A2AR Antagonists Reverse SPP1^hi^‐TAM‐Mediated Immunotherapy Resistance

As the adenosine signaling axis is a primary mechanism of immunosuppression mediated by SPP1^hi^‐TAMs, A2AR antagonists represent a rational therapeutic target. Ciforadenant is an oral, highly selective A2AR antagonist with a *K*_*i*_ value of 2.1 nM for A2AR and >1000‐fold selectivity over A1, A2B, and A3 receptors. It demonstrated significant antitumor effects in a CRPC mouse model [9]. Monotherapy with ciforadenant markedly inhibited tumor growth, reducing tumor volume by ~50% compared to the control group, primarily attributed to a decrease in the frequency and absolute number of exhausted CD8^+^ T cells. Mechanistic studies revealed that ciforadenant specifically and significantly reduced the abundance and frequency of SPP1^hi^‐TAMs (by about 60%) without affecting other myeloid subsets (e.g., classical monocytes and dendritic cells). This selective effect is likely because SPP1^hi^‐TAMs highly express ADORA2A (the A2AR gene) and depend heavily on adenosine autocrine signaling for their survival.

More importantly, ciforadenant exhibits strong synergistic effects when combined with ICIs. In a CRPC mouse model, the combination of ciforadenant and an anti‐PD‐1 antibody was superior to either agent alone, reducing tumor volume by ~80% compared to the control and leading to complete tumor regression in some mice [9]. The combination therapy increased the infiltration of multifunctional CD8^+^ T cells (producing IFN‐γ, TNF‐α, and granzyme B) and enhanced the infiltration and cytotoxicity of NK cells. Mechanistically, ciforadenant enhances the frequency of multifunctional CD8^+^ T cells by blocking adenosine signaling, while the PD‐1 inhibitor boosts T cell infiltration capacity by relieving checkpoint inhibition, resulting in synergistic augmentation of antitumor immunity at different levels. Notably, the combination therapy did not further reduce the SPP1^hi^‐TAM population, which was already significantly decreased by ciforadenant monotherapy. Instead, it altered the T cell/TAM ratio by increasing the number of effector T cells, highlighting the central role of activated T cells in the antitumor response elicited by combination therapy.

### 7.2. PD‐L1 Blockade Combined With CAR‐T Cells to Reprogram Reg‐TAMs

CAR‐engineered T cells have achieved revolutionary success in hematological malignancies (e.g., B‐cell lymphoma), but response rates in solid tumors, including PCa, remain disappointing (ORR, generally <10%). This disparity is largely attributed to the immunosuppressive TME, where Reg‐TAMs serve as a key barrier. Studies indicate that M2‐like TAMs significantly suppress CAR‐T cell proliferation and cytotoxic activity, whereas proinflammatory M1‐like TAMs do not [20]. Mechanistically, M2‐like TAMs inhibit CAR‐T cells via multiple pathways: (1) Inducible PD‐L1 expression—exposure to IFN‐γ secreted by CAR‐T cells upregulates PD‐L1 on M2‐like TAMs by 5‐ to 10‐fold, which engages PD‐1 on CAR‐T cells to deliver inhibitory signals; (2) Secretion of immunosuppressive factors—IL‐10, TGF‐β, and ARG1 deplete arginine (essential for T cell proliferation) and inhibit TCR signaling; (3) Metabolic competition—the high glycolytic activity of M2‐like TAMs competes with CAR‐T cells for glucose, leading to T cell energy exhaustion and functional impairment [20].

In a humanized PCa mouse model, although CAR‐T cell infusion initially induced transient tumor shrinkage, rapid relapse and progression occurred, closely correlated with a sharp increase in intratumoral M2‐like TAM enrichment (~3‐fold within 1 week posttreatment) [20]. Notably, CAR‐T cells themselves secrete chemokines (e.g., CCL2 and CCL5) that recruit monocytes to the TME, where they differentiate into M2‐like TAMs under the influence of IL‐10 and lactate, establishing a negative feedback loop of CAR‐T cell–M2‐like TAM mutual reinforcement. This underscores that simply increasing the CAR‐T cell dose or infusion frequency cannot overcome TAM‐mediated resistance; concurrent targeting of TAMs is essential [20].

To address this challenge, PD‐L1 blockade is combined with CAR‐T cell therapy. In a humanized PCa mouse model, PD‐L1 inhibition using atezolizumab or avelumab successfully restored CAR‐T cell‐mediated tumor killing. The combination therapy reduced tumor volume by ~60% compared to CAR‐T monotherapy and significantly decreased the number of intratumoral M2‐like TAMs (CD163^+^ and CD206^+^) [20]. Mechanistic studies revealed that the PD‐L1 blockade directly reverses CAR‐T cell inhibition and, via Fc‐mediated ADCP, clears PD‐L1‐expressing M2‐type TAMs. The critical role of the Fc‐mediated effect was confirmed using an Fc‐deficient avelumab variant (lacking the CH2 domain): while this variant restored CAR‐T cell cytotoxicity, it did not reduce M2‐like TAM numbers [20], proving that Fc‐dependent TAM clearance is a vital component of the combination’s efficacy.

Remarkably, extensive RNA sequencing analysis demonstrated that CAR‐T cells, via paracrine signaling, can reprogram residual M2‐like TAMs toward an immunostimulatory M1‐like phenotype. Conditioned media from CAR‐T cells shifted the transcriptome of M2‐like TAMs, upregulating M1‐signature genes (e.g., CD80, CXCL9, and IL1B) and downregulating M2‐signature genes (e.g., CD163, ADORA3, and IL10). This phenotypic conversion is primarily mediated by CAR‐T cell‐derived IFN‐γ and GM‐CSF: IFN‐γ activates JAK‐STAT1 signaling to induce M1 genes, while GM‐CSF upregulates MHC‐II and costimulatory molecules, enhancing antigen presentation. The reprogrammed M1‐like TAMs lose immunosuppressive function and synergize with CAR‐T cells to kill tumors—by secreting CXCL9/CXCL10 to recruit T cells, presenting tumor antigens, and producing cytotoxic molecules like iNOS‐derived nitric oxide. This immune‐activating cascade may underlie the durable antitumor immune memory observed with combination therapy as some mice rejected tumor rechallenge after initial eradication [20].

### 7.3. In Situ Reprogramming of TAMs via STING and TLR Agonist Nanodelivery

Activation of the cGAS‐STING pathway reprograms Reg‐TAMs to an immunostimulatory phenotype, as validated in multiple tumor models. STING agonists (e.g., cyclic dinucleotide cGAMP) bind STING on the endoplasmic reticulum membrane, triggering its translocation to the Golgi and recruiting TBK1 and IKK kinases to activate IRF3‐IFN and NF‐κB proinflammatory pathways [68, 70]. In TAMs, STING activation induces massive release of type I interferons (IFN‐α/β) and proinflammatory cytokines (TNF‐α, IL‐6, and IL‐12) and upregulates antigen‐presentation molecules (MHC‐II, CD80, and CD86), driving an M2‐to‐M1 phenotypic shift [69]. However, clinical translation of STING agonists faces hurdles: (1) systemic administration can cause severe cytokine storms; (2) poor bioavailability and rapid degradation by extracellular nucleases; and (3) short activity duration (hours to 1–2 days), insufficient for sustained TAM reprogramming [21, 111].

Nanodelivery systems overcome these limitations. For example, Al‐Janabi et al. [22] developed FR‐β‐targeted lipid nanoparticles (LNPs) that selectively deliver cGAMP to perivascular TAMs (which highly express FR‐β). In PCa models, anti‐FR‐β–LNP–cGAMP increased local agonist concentration by 10‐ to 20‐fold over free drug while reducing off‐target accumulation in the liver/spleen by >80% [22]. Monotherapy with this formulation significantly inhibited tumor growth and reduced M2‐like TAM infiltration (~50%) and synergized powerfully with ADT—combination therapy shrank tumor volume by ~ 70% versus ADT alone, achieving complete tumor regression and long‐term survival in some mice [22].

The synergy between STING agonists and ADT extends beyond immune activation to metabolic cross‐talk. ADT‐induced tumor cell apoptosis releases cellular DNA fragments that act as endogenous STING ligands, amplifying the cGAS‐STING pathway activation in TAMs—a “therapy‐induced immune priming” effect [22]. Furthermore, ADT shifts TME metabolism toward enhanced fatty acid oxidation and reduced glycolysis, favoring the maintenance of M1‐like TAMs (which rely on fatty acid oxidation) over glycolytic M2‐like TAMs, thus reinforcing STING‐driven reprogramming. However, ADT also upregulates immunosuppressive factors (e.g., PD‐L1 on tumor cells and Treg recruitment), which may counter STING benefits.

TLR agonists (e.g., TLR7/8 agonist resiquimod and TLR9 agonist CpG‐ODN) similarly reprogram TAMs via MyD88‐NF‐κB and TRIF‐IRF3 pathways, inducing proinflammatory cytokines and type I interferons [21]. Their stability can be improved via chemical modifications (e.g., phosphorothioate‐modified CpG), and some (e.g., imiquimod) have established clinical safety. However, like STING agonists, TLR agonists suffer from transient activity—TAM reprogramming wanes within 3–5 days postdose, allowing TME immunosuppression to recur [111, 112].

Long‐term TAM reprogramming requires sustained agonist delivery. Injectable hydrogels (e.g., calcium‐responsive alginate hydrogel loaded with STING agonist MSA‐2) form depots that release agonists slowly (over 2–3 weeks) in response to high TME calcium levels [113]. This system converted M2‐like to M1‐like TAMs in solid tumor models, significantly suppressing tumor growth. Pulsed dosing (e.g., weekly anti‐FR‐β–LNP–cGAMP) maintains reprogramming while avoiding tolerance from chronic STING activation (e.g., SOCS1 upregulation) [21].

Combination strategies enhance the durability. STING/TLR agonists plus ICIs (e.g., anti‐PD‐1/PD‐L1) reprogram TAMs and activate T cells, while checkpoint blockers prevent T cell exhaustion. In PCa models, the STING agonist + anti‐PD‐1 outperformed monotherapies, with some mice showing long‐term tumor control post‐cessation, indicating immune memory formation [22]. In bone metastases, where TGF‐β suppresses STING expression [114], adding TGF‐β neutralizing antibodies or mTOR inhibitors restores STING responsiveness, offering a path to overcome immunotherapy resistance.

### 7.4. Adjunctive Strategies for Modulating TAM Polarization: miRNA and Natural Compounds

miRNAs are endogenous posttranscriptional regulators that play a crucial role in TAM polarization and represent potential therapeutic targets. In PCa, miR‐155 exhibits a context‐dependent dual role: within M1‐like TAMs, it enhances JAK1‐STAT1 signaling by targeting SOCS1 (a suppressor of cytokine signaling), thereby promoting the expression of proinflammatory genes (e.g., iNOS and CXCL9) and driving M1 polarization [26]. However, under specific conditions such as the presence of TGF‐β, miR‐155 can promote M2 polarization by targeting SHIP1 (a negative regulator of PI3K signaling), activating the PI3K‐AKT pathway, and consequently accelerating tumor cell invasion and proliferation [115]. This context‐dependent nature necessitates precisely designed targeting strategies to avoid unintended outcomes.

The role of the let‐7 family miRNAs in regulating TAM function is well‐established. Let‐7b‐5p is significantly upregulated in M2‐like TAMs and directly targets the 3^′^UTR of SOCS1, repressing its expression. This suppression promotes macrophage polarization towards an M2 phenotype via the SOCS1‐STAT3 pathway, enhancing tumor angiogenesis and cell mobility [24, 116]. In coculture systems of PCa cells and TAMs, treatment with a let‐7b‐5p inhibitor (antisense oligonucleotide [ASO]) reversed M2 polarization, significantly reducing the secretion of VEGF and diminishing tumor cell migration capacity by ~50% [24]. Furthermore, circular RNAs contribute to the TAM function by acting as miRNA sponges. For instance, circSMARCC1, contained within exosomes secreted by PCa cells, can be transferred to TAMs. There, it sequesters miR‐1322, alleviating its inhibitory effect on CCL2 expression, which subsequently activates the PI3K‐AKT pathway, promotes M2 polarization, and facilitates tumor growth [117]. Targeting circSMARCC1 or administering miR‐1322 mimetics presents a promising new approach for TAM intervention.

Delivering therapeutic miRNAs specifically to TAMs within the TME remains a significant technical challenge. Exosomes, natural intercellular communication vesicles, offer several advantages: (1) they protect miRNAs from degradation by serum ribonucleases (RNases), enhancing stability in biological fluids [118]; (2) they exhibit low immunogenicity compared to synthetic lipid or polymer nanoparticles, reducing immune clearance; (3) they possess innate membrane fusion capabilities, enabling efficient cytoplasmic delivery of their cargo; and (4) their surfaces can be engineered with targeting ligands (e.g., against M‐CSF or folate receptor) to achieve cell‐specific targeting towards TAMs [118]. In PCa preclinical studies, engineered exosomes loaded with let‐7b inhibitors (via electroporation or transfection) successfully accumulated in intratumoral TAMs following intravenous injection, significantly reducing the expression of M2 markers (CD206 and ARG1) and inhibiting tumor growth [24].

However, exosome‐based delivery systems face challenges: (1) scalability and production: Exosome yields are low (~100 μg per 10^6^ cells), and purification processes are complex, hindering clinical translation. (2) Heterogeneity: batch‐to‐batch variations in exosome size, cargo, and surface markers can lead to inconsistent therapeutic effects. (3) Targeting specificity: Even with surface modifications, exosomes can be nonspecifically cleared by macrophages in the liver and spleen, reducing the delivery efficiency to the tumor. (4) Loading efficiency: the encapsulation efficiency for large molecules like ASOs is typically low (<5%), often necessitating high doses. Emerging technologies, such as cell‐membrane‐derived nanovesicles (produced by physical extrusion for scalable manufacture) and hybrid exosomes (fusing exosome membranes with synthetic liposomes), are being explored to overcome these limitations [118]. Before clinical application, further research is needed to fully understand the biodistribution, long‐term safety (e.g., potential for autoimmune reactions or off‐target effects), and precise immunomodulatory properties of therapeutic exosomes.

Moreover, dietary supplements and natural compounds offer a relatively safe and accessible avenue for modulating the TME. Omega‐3 polyunsaturated fatty acids, such as EPA and DHA, have demonstrated anti‐TAM effects in PCa studies through multiple mechanisms: (1) they competitively inhibit the metabolism of omega‐6 fatty acids (e.g., arachidonic acid), reducing the production of proinflammatory lipid mediators like prostaglandin E2 and leukotriene B4; (2) their metabolites (e.g., resolvins and protectins) activate the G‐protein coupled receptor GPR120, inhibiting NF‐κB nuclear translocation and STAT3 activity; and (3) they inhibit the maturation of SREBP1, reducing the expression of FASN and thereby blocking lipid accumulation in LAMs/SPP1^hi^‐TAMs. In mouse models of castration‐resistant PCa, a diet high in omega‐3 significantly reduced intratumoral TAM infiltration and M2 marker expression while downregulating prometastatic genes (e.g., ARG1, CSF‐1R, MMP9, VEGF, and CCL2) [85, 119]. Epidemiological studies also suggest that high omega‐3 intake is associated with a lower risk of PCa incidence and progression, particularly in patients undergoing ADT [25].

Bioactive compounds derived from traditional medicine also show potential for modulating TAMs. For example, tanshinone I inhibits the tyrosine 705 phosphorylation of STAT3, reducing its translocation to the nucleus and subsequent IL‐6‐mediated CCL2 secretion, thereby inhibiting TAM recruitment and M2 polarization. In coculture systems of PCa cells and TAMs, tanshinone I treatment decreased M2 markers (CD163 and IL‐10) and increased M1 markers (iNOS and TNF‐α), suppressing tumor cell proliferation and migration. Flavonoids like kaempferol and quercetin can stimulate the release of GM‐CSF from PC‐3 PCa cells, potentially activating dendritic cells and enhancing the host’s adaptive immune response [120, 121].

The advantages of natural compounds include their favorable side‐effect profiles, good patient compliance, and potential as preventive measures for high‐risk individuals. However, clinical translation faces hurdles: (1) low bioavailability: Oral absorption for many natural compounds is less than 10%, often requiring high doses or formulation improvements (e.g., nanoformulations and phospholipid complexes). (2) Pleiotropic effects: Their action on multiple targets can lead to both synergistic benefits and unintended off‐target effects, necessitating detailed pharmacological studies. (3) Insufficient clinical evidence: Most evidence remains at the cellular and animal model stages, lacking support from high‐quality randomized controlled trials. A more realistic translational path may involve using natural compounds as adjuncts or maintenance therapy in combination with standard treatments (e.g., ADT) or immunotherapy.

## 8. Current Challenges and Future Research Directions

### 8.1. Current Challenges

The core challenges in current PCa TAM research lie in their extreme complexity and obstacles to therapeutic translation. At the fundamental research level, while the functional heterogeneity of TAMs (e.g., SPP1^hi^‐TAMs and LAMs) has been revealed by single‐cell technologies, the dynamic transitions between subsets, their spatial distribution, and intercellular communication networks remain unclear [9], necessitating spatiotemporal multidimensional analysis (e.g., spatial transcriptomics) for clarification. Mechanistically, TAMs mediate immunosuppression through multiple redundant pathways, such as adenosine and PD‐1/PD‐L1, requiring the development of multitarget combination strategies (e.g., A2AR antagonist + PD‐1 inhibitor), which increases the difficulty of toxicity management [9, 122]. Regarding therapeutic strategies, although agents like STING/TLR agonists can effectively reprogram TAMs, their activity is short‐lived (<3 days), and current nanodelivery systems face bottlenecks such as poor tumor penetration, low endosomal escape efficiency (<10%), and unknown long‐term safety [21, 111, 112]. CAR‐T therapy, on the other hand, suffers from limited solid tumor infiltration, poor in vivo persistence, high costs of scaled‐up production, complexities of combination therapy toxicity (e.g., CRS), and unclear optimal sequencing with drugs like PD‐L1 inhibitors [20]. In biomarker translation, predictive indicators like SPP1^hi^‐TAM lack standardized detection methods suitable for FFPE tissues (e.g., multiplex immunofluorescence) and validated clinical thresholds. Regarding target toxicity, blockade of the CD47‐SIRPα axis can cause severe anemia, necessitating the development of tumor‐selective bispecific antibodies or conditionally activated antibodies to improve the therapeutic window [122, 123]. Finally, existing preclinical models (e.g., xenograft models and PDX) inadequately recapitulate the complexity of the human TME, limiting their clinical predictive value [92].

### 8.2. Future Research Directions

Therefore, the future direction of TAM‐targeted therapy for PCa must focus on multidimensional collaborative innovation. At the cellular therapy level, enhancing CAR‐T cell infiltration into solid tumors (especially bone metastases) can be achieved by combining vascular normalization drugs or engineering chemokine receptor expression (e.g., CXCR4). Utilizing fourth‐generation or armored CAR‐T cells (e.g., expressing a dominant‐negative TGF‐β receptor) can improve their adaptability and persistence within the immunosuppressive TME. Simultaneously, advancing universal CAR‐T or in vivo CAR‐T generation technologies is needed to address the bottlenecks of scaled production and cost, though optimal dosing sequences and toxicity management strategies (e.g., for CRS) in combination with PD‐L1 inhibitors require clarification in well‐designed clinical trials [20]. In terms of treatment monitoring, developing noninvasive dynamic assessment based on liquid biopsies is crucial, focusing on changes in levels of TAM‐secreted soluble factors (e.g., sSPP1, CCL2, CXCL5, and IL‐6) and TAM‐derived exosomes (e.g., carrying PD‐L1 and TREM2) to predict efficacy early [4, 6, 67]. In the preclinical research field, reliance on advanced models like patient‐derived “immune organoids” and three‐dimensional microfluidic chips (tumor‐on‐a‐chip), particularly systems capable of mimicking the bone metastasis microenvironment, will provide specific models for personalized drug screening [15, 91]. A fundamental shift lies in moving from a “single‐target” to a “holistic network” perspective, utilizing single‐cell multiomics combined with spatial omics (e.g., Visium and CODEX) and computational modeling to systematically deconvolute the interaction networks between TAMs, tumor cells, and other immune and stromal components [9, 32]. This will lay the foundation for achieving precise, personalized immune modulation—matching combination regimens to the dominant TAM subset characteristics within a patient’s tumor. Ultimately, this represents a paradigm shift in therapeutic philosophy: from merely killing tumor cells to systemically remodeling the TME, from monotherapy to multitarget combination regulation, and from a “one‐size‐fits‐all” approach towards true precision medicine.

## 9. Conclusion

In conclusion, TAMs play a pivotal role in driving the immunosuppressive TME and the progression of PCa to advanced stages. Future research should prioritize therapeutic strategies that precisely target TAM subsets to overcome immunotherapy resistance, alongside addressing challenges such as TAM heterogeneity, drug delivery optimization, and biomarker development for patient stratification. Ultimately, advancing combination therapies that simultaneously target tumor cells and remodel the immunosuppressive microenvironment will be essential for improving clinical outcomes.

## Author Contributions

Conceptualization: Si Shen and Zhonghao Tang. Methodology: Chen Guo. Investigation: Yuanyuan Mi. Writing – original draft preparation: Si Shen, Zhonghao Tang, and Chen Guo. Writing – review and editing: Yuanyuan Mi.

## Funding

This work was supported by the National Natural Science Foundation (Grant 81802576), the Wuxi Commission of Health and Family Planning (Grant M202330), the Talent Plan of Taihu Lake in Wuxi (Double Hundred Medical Youth Professionals Program) from the Health Committee of Wuxi (Grant BJ2023051), and the Jiangsu Province 7th phase “333” high‐level talents.

## Disclosure

All authors have read and approved the final manuscript.

## Conflicts of Interest

The authors declare no conflicts of interest.

## Data Availability

No datasets were generated or analyzed during the current study.
